# The effect of polishing protocol on surface gloss of different restorative resin composites

**DOI:** 10.1080/26415275.2019.1708201

**Published:** 2020-01-03

**Authors:** Lippo Lassila, Eija Säilynoja, Roosa Prinssi, Pekka K. Vallittu, Sufyan Garoushi

**Affiliations:** a Department of Biomaterials Science and Turku Clinical Biomaterials Center – TCBC, Institute of Dentistry, University of Turku, Turku, Finland; b Research Development and Production Department, Stick Tech Ltd – Member of GC Group, Turku, Finland; c City of Turku Welfare Division, Oral Health Care, Turku, Finland

**Keywords:** Resin composite, surface gloss, polishing protocol

## Abstract

**Aim:**

The purpose of this *in vitro* study was to determine the effects of different polishing protocols on the surface gloss (SG) of different commercial dental resin composites (RCs).

**Material and methods:**

A total of 147 block-shaped specimens (40 mm length × 10 mm width × 2 mm thick) were made from conventional RCs (G-aenial Ant. and Flo X), bulk-fill RC (Filtek Bulk Fill), fluoride-releasing RCs (BEAUTIFIL II, ACTIVA-Restorative) and discontinuous microfiber-reinforced RCs (Alert and everX Flow). Each group was subdivided into seven subgroups (*n* = 3), according to polishing protocol: Laboratory-machine polishing with different siliconcarbide paper grits (G1: 320) → (G2: 800) → (G3: 1200) → (G4: 2000) → (G5: 4000). Chairside-hand polishing using a series of Sof-Lex spiral (G6) and abrasive polishing points (G7). Glossmeter was used to determine the SG at 60° incidence angle. SG was measured before and after polishing. Three-dimensional (3 D) noncontact optical profilometer and scanning electron microscopy (SEM) analysis were performed. Data were analyzed using ANOVA (*p* = .05).

**Results:**

Significant differences in SG (ranged 3–93 GU) were found according to the type of polishing protocol and RC (*p* < .05). Specimens polished with 4000 grit paper showed the highest SG (93 GU) values among all the groups tested.

**Conclusions:**

The tested chairside-hand polishing protocols presented lower SG values than laboratory-machine polishing (4000 silicon paper grit) and unpolished surfaces.

## Introduction

Esthetic concepts have been particularly important in driving the development of restorative resin composites (RCs) in the last few years. A glossy and perfectly smooth surface is a requirement for a desirable esthetic appearance [1]. It also needs to remain like this for a long period within the oral environment. The smooth surface, apart from enhancing the esthetic result, prevents the formation of discoloring films and plaque retention due to the absence of micro-roughness [1]. Moreover, surface smoothness decrease the coefficient of friction and subsequently, this may reduce wear rate [2], which compromises the clinical performance of the composite restorations. In recent years, restorative RCs have rapidly evolved both in terms of filler particles and resin matrix composition and structure. The application of nano, bulk-fill, fiber-reinforcement and ion-releasing technologies in the dental materials field has resulted in the development of new RCs containing different size and shape particles [3–5]. These materials incorporate a different volume fraction of filler particles, with different sizes ranging from micrometer to nanometer scale. Manufacturers claim that the characteristics of these materials include improved handling properties, adequate strength and high gloss ceramic-like polished surface which mirror the natural enamel and dentin. However, independent *in vitro* and *in vivo* studies, especially on the esthetic appearance of these RCs, in terms of their surface gloss (SG) is limited.

Gloss is an important property and is used primarily as a measure of surface shine [6]. The gloss of a surface may be defined as its degree of approach to a mirror surface. A perfect mirror surface is said to have maximum gloss [6]. It has been suggested that SG can be determined by both the intrinsic characteristics of the RC and the finishing and polishing procedures [7]. Thus, a successful composite restoration requires not only care for restorative material selection, with ideal esthetics and mechanical strength characteristics, but also care with respect to the choice of the finishing and polishing protocol [1,8]. Several finishing and polishing protocols are available on the market, including diamond burs, rubber cups, discs and abrasive pastes [8–10]. Many researchers have studied the polishability of different polishing protocols on the surfaces of various commercial RCs [8–14]. Some studies have indicated that aluminum oxide disks produce smoother surfaces when compared with diamond burs, tungsten carbide drills and rubber cups associated with polishing pastes [11–14]. Usually, the polishing protocols have been evaluated according to the manufacturers’ recommendations. However, the manufacturers rarely support their recommendations with objective investigations that have proven the suggested protocol to be superior to others. Therefore, it would be helpful to compare the clinical polishing protocols with sequences of standardized laboratory-machine polishing protocols to supply quantitative proof for the suggested procedure.

Nevertheless, very few studies have investigated the influence of different surface polishing protocols on the SG of a new commercial RCs used for direct restorations. Thus, the aim of this *in vitro* study was to determine the effects of different polishing laboratory-machine and clinical protocols on the SG of a seven different commercial RCs (conventional, bulk-fill, fluoride-releasing and discontinuous microfiber-reinforced). The null hypotheses were that no difference in SG would be found among the polished RCs or among the different polishing protocols when used on the same RCs.

## Materials and methods

A total of 147 block-shaped specimens (40 mm length × 10 mm width × 2 mm thick) of seven different commercial RCs (Table 1), were made in half-split molds between transparent Mylar sheets. A thin glass plate was placed on the RC free surface to remove the material excess. Polymerization of the RC was done using a hand light-curing unit (Elipar TM S10, 3M ESPE, Germany) for 20 s in ten separate overlapping portions from one side of the mold. The wavelength of the light was between 430 and 480 nm and light intensity was 1600 mW/cm^2^. The specimens from each RC were then divided into seven groups (*n* = 3) according to the polishing protocols. Laboratory-machine polishing was performed using different silicon paper grit sizes (G1: 320) → (G2: 800) → (G3: 1200) → (G4: 2000) → (G5: 4000) at 300 rpm while water-cooled using an automatic polishing machine (Struers Rotopol-11, Copenhagen, Denmark) for 1 min. Chairside-hand polishing was performed using a series of Sof-Lex spiral (beige and pink, G6) (3M ESPE, St Paul, MN) and abrasive polishing points (yellow, G7) (Jiffy composite polishers, Ultradent Products, Inc., South Jordan, UT) with a low-speed handpiece (12,000 rpm) underwater cooling and with constant moving repetitive stroking action. One side of the specimen surface facing the mold was polished and this side was named as the polished side. The other side of the specimens facing the glass slide and mylar strip remained unpolished and was named the unpolished side. After polishing, specimens were rinsed with water and stored dry at room temperature before testing.

**Table 1.** The investigated materials and their composition.

Material (shade)	Manufacturer	Matrix composition	Inorganic filler content
ACTIVA-Restorative (A2)	Pulpdent Corp, Watertown, USA	Blend of diurethane and other methacrylates with modified polyacrylic acid	55.4 wt% Silica, bioactive glass and sodium fluoride fillers (Ø NR)
G-aenial Anterior (A3)	GC Corp, Tokyo, Japan	UDMA, dimethacrylate co-monomers	76 wt% Pre-polymerized filler (Ø 16–17 µm), silica and strontium fluoride containing fillers (Ø > 100 nm)
everX Flow (Dentin shade)	GC Corp, Tokyo, Japan	Bis-EMA, TEGDMA, UDMA	70 wt% Short glass fiber (Ø 6 µm & barium glass fillers Ø 700 nm)
Filtek Bulk Fill (A2)	3M/ESPE, St. Paul, MN, USA	AUDMA, UDMA, DDDMA	76.5 wt% Zirconia/silica and ytterbium trifluoride fillers in nanometer scale (av. Ø 20 nm)
Alert (A3)	Jeneric/Pentron, Wallingford, CT, USA	Bis-GMA, UDMA, TEGDMA, THFMA	84 wt% Silica (Ø 800 nm) and micrometer scale glass fiber (Ø 7 µm)
G-aenial Flo X (A3)	GC Corp, Tokyo, Japan	UDMA, dimethacrylate co-monomers	69 wt% Barium glass fillers in nanometer scale (av. Ø 700 nm)
BEAUTIFIL-II (A3)	Shofu Inc., Kyoto, Japan	Bis-GMA, UDMA, Bis-MPEPP, TEGDMA.	83.3 wt% Fluoro-silicate glass (av. Ø 800 nm)

Bis-GMA: bisphenol-A-glycidyl dimethacrylate; TEGDMA: triethylene glycol dimethacrylate; UDMA: urethane dimethacrylate; AUDMA: aromatic urethane dimethacrylate; DDDMA: 12-dodecanediol dimethacrylate; Bis-EMA: ethoxylated bisphenol-A-dimethacrylate; Bis-MPEPP: bisphenol A polyethoxy methacrylate; THFMA: tetrahydrofurfuryl-2-methacrylate; wt%: weight percentage; NR: not reported.

In total, there were seven groups (*n* = 3) per each material involving five different laboratory-machine polishing paper grits and two chairside-hand polishing protocols. The SG was assessed for polished and unpolished sides.

The SG was measured at 60° incidence angle, using a calibrated infrared Zehntner-Glossmeter (GmbH Testing Instruments, Darmstadt, Germany) with a square measurement area of 6 mm × 40 mm area. The average of five measurements was recorded per surface.

Three-dimensional (3D) noncontact optical profilometer (Bruker Nano GmbH, Berlin, Germany) using Vision64 software was used to observe and capture images of the polished surfaces (*n* = 3) from each of the polishing protocols.

Scanning electron microscopy (SEM, GeminiSEM 450, Carl Zeiss, Oberkochen, Germany) provided the characterization of the microstructure of the investigated RCs. Polished specimens (4000 grit) from each material (*n* = 2) were stored in desiccator for 1 d. Then, they were coated with a gold layer using a sputter coater in vacuum evaporator (BAL-TEC SCD 050 Sputter Coater, Balzers, Liechtenstein) before the SEM examination. SEM observations were carried out at an operating voltage of 5 kV and working distance of 3–6 mm.

The data were analyzed using SPSS version 23 (SPSS, IBM Corp., Armonk, NY) using analysis of variance (ANOVA) at the *p* < .05 significance level followed by a Tukey HSD *post hoc* test to determine the differences between the groups.

## Results

**Figure 1.**
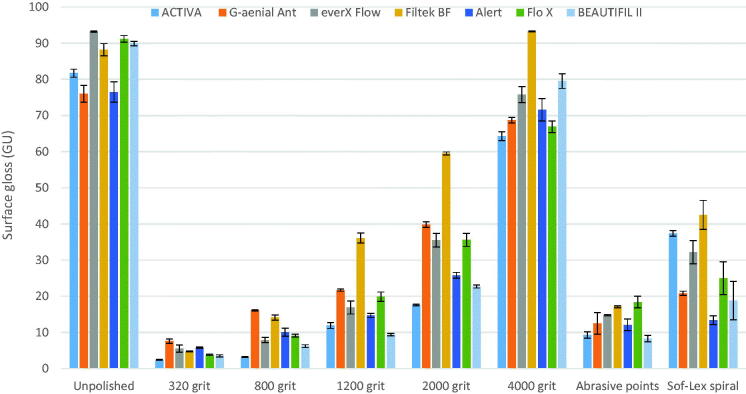
Surface Gloss (GU) mean values of specimens in relation to different polishing protocols. Vertical lines represents standard deviation.

**Figure 2.**
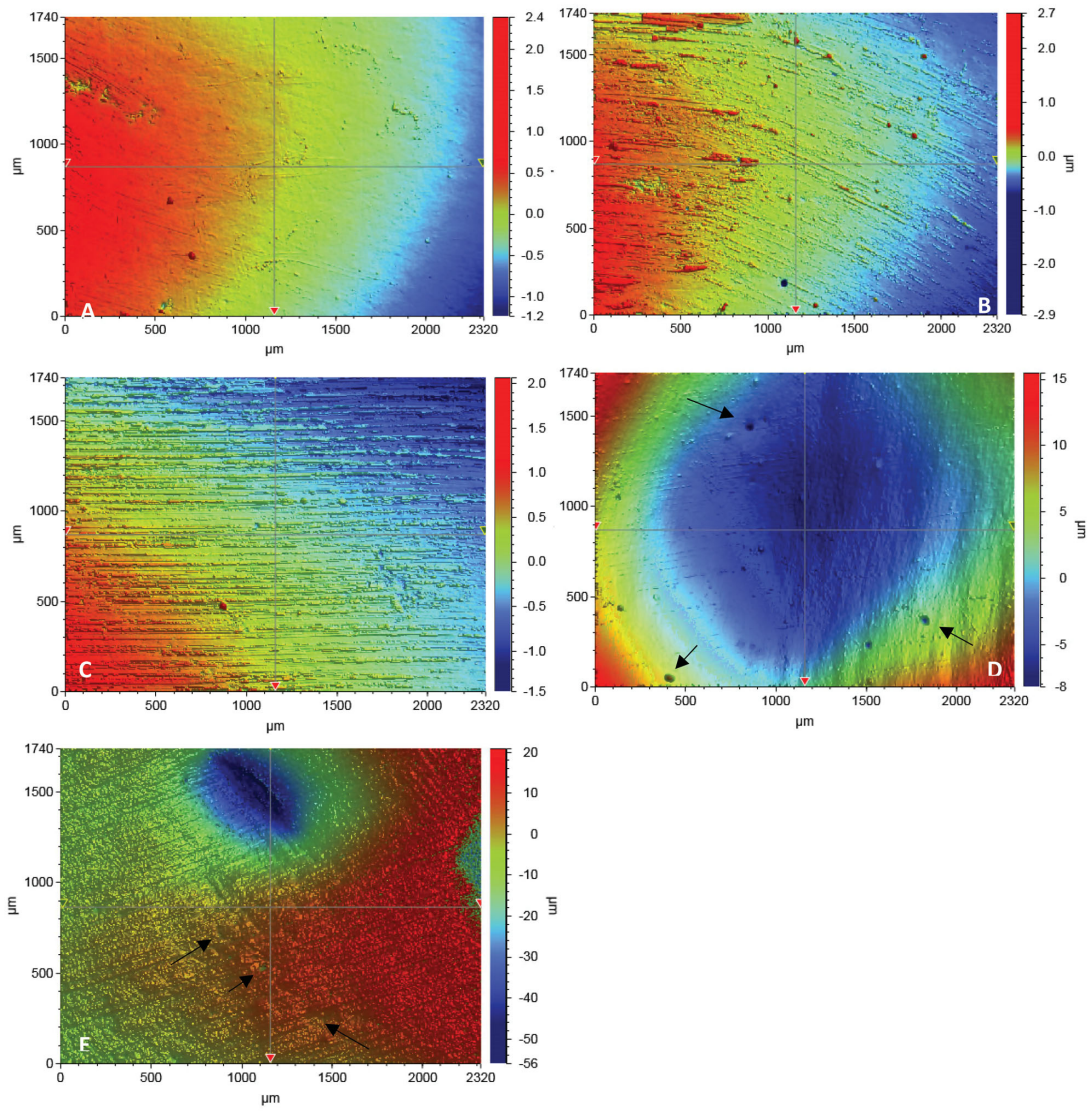
Typical 3 D surface profile of specimens in relation to different polishing protocols. A: 4000; B: 2000 grit; C: 1200 grit; D: Sof-Lex spirals; E: Abrasive points. Arrows indicate small pit defects.

The SG mean values of the tested RCs after various polishing protocols are shown in Figure 1. Significant differences in SG (ranged 3–93 GU) were found according to the type of material and polishing protocol (*p* < .05), however, some interaction existed between the groups. The polishing protocol was always a significant and stronger factor than the type of material. Specimens polished with 4000 grit paper showed significantly the highest SG (93 GU) values among all the groups tested. As seen in Figure 1, the lowest SG values were observed for specimens polished by 320 grit paper. All polishing protocols (except two RCs polished with 4000 grit) used in this study decreased the SG of composites surfaces in comparison to the unpolished surfaces (*p* < .05). SG after polishing with Sof-Lex spiral was significantly higher than surfaces polished by abrasive polishing points (*p* < .05), regardless of the material used. In four of the seven polishing protocols, Filtek Bulk Fill composite presented a higher SG values than did the other tested RCs. The 3D images after each polishing protocol are shown in Figure 2. Wide scratches and a large amount of small pits resulting from filler particle exfoliation were seen after using chairside-hand polishing protocols (Figure 2(D,E)). Shallow scratches were observed on the surface of specimens following polishing with 1200 and 2000 grit paper (Figure 2(B,C)). However, after polishing with 4000 paper grit, the scratches disappeared and surfaces became uniform and smooth (Figure 2(A)).

**Figure 3.**
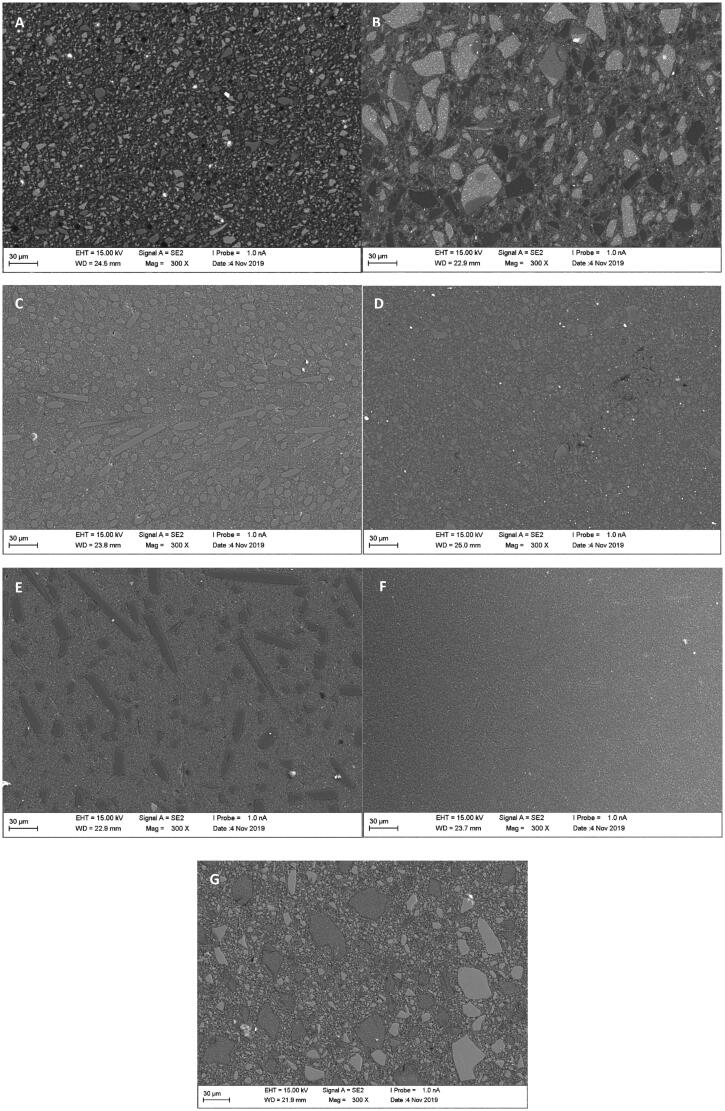
SEM photomicrographs of polished surface (4000 grit) of investigated materials. (A) Activa; (B) G-aenial Ant; (C) everX Flow; (D) Filtek BF; (E) Alert; (F) Flo X; (G) BEAUTIFIL-II.

SEM analysis showed typical microstructure of each tested material with different particulate fillers size and shape in polymer matrix (Figure 3). This suggested an explanation for different SG behaviors between tested materials.

## Discussion

In this study, seven restorative RCs were selected based on different filler concepts in each material. The SG investigated after being polished using different polishing protocols. Both the type of material and the polishing protocols significantly affected the SG values and hence the null hypotheses were rejected. High gloss for a RC gives a natural, esthetic appearance to a restoration. According to ISO semigloss surfaces like RCs should be measured with 60° angle of illumination, which was applied in this. This was found closer to the angle from which the average person will observe the surface [15]. The high filler loading of nanosized particles (av. Ø 20 nm) in Filtek Bulk Fill results in higher SG values than did most of the other tested RCs. This observation supported by previous investigations on the effect of filler size on gloss of RCs [13,16]. However, some other studies have shown that factors such as monomer type, degree of monomer conversion and refraction index can also influence the gloss of RCs [17,18]. Discontinuous microfiber-reinforced RCs (Alert and everX Flow) showed comparable SG values than other tested conventional (G-aenial Ant. and Flo X) and fluoride-releasing (BEAUTIFIL II, ACTIVA-Restorative) RCs. The polished surfaces of these microfiber-reinforced RCs were relatively smooth, similar to that of particulate filled RCs used (Figure 3). The protrusion of microfibers was not observed and instead of the fibers being pulled out to produce a pitted surface, the fibers were polished down together with resin matrix. It should be taken into account that microfiber-reinforced RC (everX Flow) is instructed to be used as bulk base or core foundation and should not be used as top surface layer. It should be covered with a layer of conventional RC to ensure sufficient esthetic appearance.

Gloss reduction of RCs following brushing test was observed in previous studies [19,20]. The decrease in gloss was attributed to increased roughness and change in surface topography resulting from abrasion of resin matrix and loss of surface filler particles. According to Bayne et al., the amount of filler is not as important as its pattern of dispersion and the inter-particle spacing of filler particles plays a vital role in composite surface protection [21]. In another study by Valente et al., the higher SG of the submicron or nanofilled (av. Ø 175 nm) composite both before and after brushing abrasion suggested that smaller inorganic filler particles could be advantageous in retaining superior esthetic properties following exposure to oral environment [22].

Many researchers showed the correlation between SG and surface roughness [15,23–25]. The lower the surface roughness the higher the SG. Heintze et al. reported that the SG improved consistently during the polishing procedures [23]. But researchers also reported that the improvement of surface roughness was not similar to the improvement of SG, and differed from material to material [8,26]. In general, it has been stated that when the surface roughness is increased, decreased gloss occurs [1].

As recommended by Roeder et al., in our study we measured the SG of materials before and after polishing to homogenize the specimens [27]. It was observed that the SG of the RCs against the Mylar sheet (unpolished surface) was significantly higher than after polishing with chairside-hand protocols (Figure 1). Similar results can also be observed in the studies of Hoelscher et al., Lassila et al. and Cazzaniga et al. [14,25,28]. Although surfaces light-cured against a Mylar sheet are smoother and glossy, in most cases finishing of the restoration is necessary to remove excess material and to recontour; this reduces the surface glossiness and necessitates restoration polishing [29]. Moreover, the polymerized surface against the Mylar sheet is rich in resin matrix (oxygen inhibition layer) and is less resistant to abrasion and can contain bubbles [30].

By comparing SG values obtained with different polishing protocols, it can be clearly observed that laboratory-machine polishing with 2000 and 4000 silicon paper grits obtained glossier surfaces than chairside-hand polishing protocols (Figure 1). In addition, for most RCs Sof-Lex spiral resulted in a significantly glossier surface than did polishing with abrasive polishing points. Consistent with our study, Pala et al. reported that multistep systems (Sof-Lex spiral) produced higher gloss, while the one-step system (abrasive polishing point) produced the lowest gloss [8]. These differences in results can be explained by the hardness and type of the abrasive, and the geometry of the instruments employed [31]. According to Blank, the design of Sof-Lex spiral wheels employ 2 parallel rows of 15 individually radiating elastomeric '‘bristles’ uniformly impregnated with abrasives [32]. The flexible form can adapt to nearly every surface of a restoration, minimizing heat formation and unwanted pressure during polishing. Several studies concluded that flexible aluminum discs are the best instruments for producing the surface glossiness [8,33]. However, one could also recommend the abrasive polishing points, since points may be used clinically in areas that are not readily accessible to other polishing systems.

According to the American Dental Association (ADA) professional product review, 40–60 GU was identified as a typically desired gloss based on observations from an expert panelist [34]. Cook and Thomas reported that poor polish is generally considered to be below 60 GU, with an acceptable polish being between 60 and 70 GU [35]. According to this, only the laboratory polishing protocol up to a 4000 grit size used in the study exhibited successful gloss results. In this study, the 3D optical profilometer results (Figure 2) were consistent with the SG results. Surface profile observations revealed that deeper and more frequent scratch lines (irregularities) were evident for the rough silicon paper grits and chairside-hand polishing protocols.

The limitation of this study is that specimen preparation was done by two investigators and this might have had an effect on the pressures exerted during the polishing procedures although the polishing time was controlled. A negative control group of RC whose roughness was provided using a diamond finishing bur on the surface is missing and this will be evaluated in the near future.

## Conclusion

According to the research methodology used, the SG of evaluated RCs is influenced by the polishing protocol used. The smoothest and most glossy surfaces were obtained with laboratory-machine polishing protocol (4000 grit).
